# Marine Hospital for Baltimore

**Published:** 1880-06

**Authors:** 


Marine Hospital for Baltimore.—We are pleased to learn that efforts are being made to procure funds from Congress for the establishment of a Marine Hospital for this city. When the population, (about 400,000) and the extent of the commerce of Baltimore, (second only to that of New York) are considered, it is surprising to learn that in fact there is no Marine Hospital attached to the port at all. Seamen having been cared for, until now, in private hospitals by contract! The rapid increase in the commerce of Baltimore within the past decade, would seem to render a large, commodious and eligibly located hospital for the treatment of sick and disabled seamen a *necessity* at the present time, and as such no difficulty would seem likely to intervene in procuring an appropriation sufficient to erect and equip, an establishment that would be creditable alike to Baltimore and the country at large.

